# Antidote for Poisons

**Published:** 1876-06

**Authors:** 


### *A NEW GENERAL ANTIDOTE FOR POISONS.*

M. Jeannel gives the following formula for an antidote for a number of deadly poisons: Solution of sulphate of iron 0.145) 100, water 800, calcined magnesia 80, washed animal charcoal 40. These ingredients are kept separate, the solution of sulphate of iron in one vessel, the magnesia and charcoal in another, with some water. When needed, the sulphate solution is poured into the last mentioned receptacle and violently agitated. The mixture should be administered promptly in doses of from 1-6 to 3-3 ounces. From experiments M. Jeannel finds that this antidote, employed in proper proportions, renders preparations of arsenic, zinc, and digitaline completely insoluble. It does not render oxide of copper absolutely insoluble, however, and leaves in solution notable quantities of morphine and strychnine. It neither decomposes nor precipitates cyanide of murcury nor tartar emetic. It saturates free iodine entirely, and acts but partially upon solutions of alkaline hypochlorites. Four ounces of the antidote are found to neutralize the poisonous effect of 1-6 ounces of arsenite of soda. It retards the toxic action of sulphate of strychnin, affording sufficient delay to administer evacuants. One third of an ounce is efficacious against digitaline injected into the intestines. The formula, says M. Jeannel, is certainly preferable to the official hyprated peroxide of iron, which, in course of time and at a temperature of 59° Fahr.,undergoes molecular modifications which render it unreliable as an antidote for arsenical preparations.

